# Crack Evolution and Oxidation Failure Mechanism of a SiC-Ceramic Coating Reactively Sintered on Carbon/Carbon Composites

**DOI:** 10.3390/ma14247780

**Published:** 2021-12-16

**Authors:** Min Xu, Lingjun Guo, Hanhui Wang

**Affiliations:** State Key Laboratory of Solidification Processing, Carbon/Carbon Composites Research Center, Northwestern Polytechnical University, Xi’an 710072, China; xumin@mail.nwpu.edu.cn (M.X.); wanghanhui@mail.nwpu.edu.cn (H.W.)

**Keywords:** carbon/carbon composites, computed tomography, SiC ceramic coating, failure detection, crack

## Abstract

A SiC ceramic coating was prepared on carbon/carbon composites by pack cementation. The phase composition and microstructure of the coated specimens were characterized using X-ray diffraction instrument and scanning electron microscope. The results showed that the mass-loss percentage of the coated specimen was 9.5% after being oxidized for 20 h. The oxidation failure of the SiC ceramic coating at 1773 K was analysed by non-destructive X-ray computed tomography. The effective self-healing of cracks with widths below 12.7 μm introduced during the coating preparation process and generated while the specimens cooled down from the high oxidation temperature prevented the oxidation of carbon/carbon composites. X-ray computed tomography was used to obtain three-dimensional images revealing internal damage caused by spallation and open holes on the coating. Stress induced by heating and cooling caused the formation, growth and coalescence of cracks, which in turn led to exfoliation of the coating and subsequent failure of oxidation protection.

## 1. Introduction

Carbon-fibre-reinforced carbon matrix (C/C) composites are extensively applied in aerospace [1,2] for unique properties of low density, high strength retention at elevated temperatures, and good ablation resistance associated with a low thermal expansion coefficient (CTE) [3,4]. However, the poor oxidation resistance of C/C composites results in severe degradation of their mechanical properties, which is a fatal flaw precluding long-term use in elevated temperature environments [5].

Coatings have been demonstrated to provide outstanding protection against oxidation [6,7,8,9,10,11,12,13]. Ultrahigh temperature ceramics (UTCs), such as TaC, ZrC and SiC, are the first choice for anti-oxidation coatings. Among numerous available UTCs, SiC is considered an ideal coating material for oxidation protection because of a high decomposition temperature (2818 K), chemical inertness and a low CTE. In addition, the thermal oxidative reaction product of SiC (silica) has good self-healing ability and low oxygen diffusivity, which are desirable for protecting C/C composites. Techniques have been proposed for preparing SiC ceramic coatings, such as thermal chemical vapour deposition (CVD), pack cementation (PC), slurry brushing and plasma spray [14,15,16,17]. Among these techniques, PC is very attractive for facile operation, low cost and good coating/substrate bonding strength. However, when the coatings are allowed to cool from the preparation temperature (commonly above 2273 K) to room temperature, residual thermal stress inevitably induces microcracks in the PC coating that deteriorate the oxidation performance of SiC [18]. Many attempts have been made to promote the oxidation resistance of SiC ceramic coatings. Kim [19] studied the thermal stress and oxidation-resistance performance of a C-SiC functional gradient coating prepared using low-pressure CVD technology. Miranda’s team developed a method to deposit gradient SiO_2_-SiC coatings on C/C substrates [20]. Hu [21] and Qiang [22] reported the oxidative resistance of a modified SiC coating. Li and coworkers performed a comparative study on the oxidative behaviour and coating microstructure of two SiC ceramic coatings produced by PC and CVD [23]. Huang et al. [24] observed the oxidation properties of SiC ceramic coatings fabricated using various processing parameters. Zhu et al. [25] fabricated a compound glass coating on SiC-coated C/C composites by slurry and pre-oxidation method. The coating provided C/C composites against oxidation at 1773 K for 447 h and 1973 K for 45 h. Li et al. [26] prepared a ZrB_2_-rich compact transition layer by slurry painting and heating carbonization method to improve the oxidation resistance of SiC-Si coating, and the bending strength of coated samples was investigated. The results that the mass-gain percentage was 1.03% at 1773 K for 342 h illustrated the outstanding oxidation resistance. The main focus of the aforementioned study was the preparation, microstructure and oxidation properties of coatings. Destructive testing methods have been employed in most studies to determine the failure mechanism of oxidation coatings [27,28,29]: for example, the cutting and polishing involved in preparing a cross-section inevitably damages morphological integrity. Moreover, performing scanning electron microscopy on a cross-section only provides limited information, because all internal failure sites cannot be observed directly. X-ray computed tomography, by contrast, can be used to visualize all sites and the complete morphology of internal oxidation damage for C/C composites and provide three-dimensional images of coating specimens.

Crack self-healing in SiC has been reported by many researchers. Petrovic and Jacobson [30] investigated flaw healing and the influence of the annealing environment on the fracture stress. In the aforementioned study, flaws were produced by Knoop microhardness indentation. Korouš et al. investigated the oxidative healing behaviour of cracks in three different commercial SiC ceramics, into which flaws were introduced into the samples using a Vickers indentor [31]. Chu [32] studied the healing of through-cracks introduced into samples by Vickers indentation. Lange [33] investigated the healing of surface cracks produced by quenching specimens in water. In the aforementioned study, pre-crack healing of the specimen surface was observed. However, studies have not been performed on original cracks formed during preparing coating preparation and new cracks generated during oxidation and cooling from high temperatures. To elucidate the microstructure evolution and failure mechanism of a coating during environmental thermal cycling from high to low temperatures, it is necessary to investigate the healing behaviour of original and newly produced cracks.

In this study, a SiC ceramic coating was first prepared on a C/C substrate by reactive sintering. Sealing of both original and newly produced cracks in SiC coatings were observed. Subsequently, X-ray computed tomography, a non-destructive test, was applied to investigate the oxidation failure of the SiC coating. The failure mechanism of the coated specimen was analysed.

## 2. Experimental Procedures

### 2.1. Specimen Preparation

Rectangular specimens (10 × 10 × 5 mm^3^) were cut from chemical-vapour-infiltrated bulk C/C composites with an apparent density of 1.72 g/cm^3^. The process for infiltrating C/C composites has been detailed previously [34]. The specimens were manually ground using 400-mesh SiC sandpapers. The specimens were then cleaned ultrasonically in analytical pure ethanol and dried at 373 K for 3 h. Subsequently, PC was used to coat the dry specimens with reactively sintered SiC. The raw material powder used to produce the reactively sintered SiC coating was composed of [28] 60–80 wt.% Si, 5–15 wt.% α-Al_2_O_3_ and 15–25 wt.% natural graphite. The powder was milled until uniformly blended. The mixed powder was transferred to a high-purity graphite crucible, and the dry specimens were wrapped in the powder. Finally, the crucible was heated at 2000–2400 K in argon for 2 h, and the power to the electric furnace was then shut down to allow the specimens to cool to room temperature naturally.

### 2.2. Oxidation Tests

To investigate the isothermal oxidation property, the coated specimens were oxidized in an electric heating furnace at 1773 K in static air. The prepared coated specimens were placed in the chamber of the furnace, and the furnace temperature was maintained for a set time. Then, the specimens were allowed to rapidly cool to the temperature of the ambient atmosphere. The cooled specimens were weighed using a high-sensitivity (0.1 mg) electronic balance. The mass loss percentage (Δw%) of a coated specimen was calculated by the following equation:

$$\Delta w\%=\frac{m_{0}{\;–\;m}_{1}}{m_{0}}\;\times \;100\%$$

where *m*_0_ and *m*_1_ are the mass of the specimen before and after oxidation, respectively.

### 2.3. X-ray Computed Tomography

High-resolution scanning of the coated specimen after oxidation was performed using a YXLON Y. Cheetah X-ray computed tomography instrument. The source voltage of the equipment was 90 kV, and the source current was 31.1 μA. During the scan, the specimen was rotated in the X-ray beam over 360° in 0.5° steps. The spatial resolution of the 3D image was 11 μm per voxel. VgStudioMax3.0 software was used to postprocess the images.

### 2.4. Phase Characterization

The phase composition of the coated specimens before and after oxidation was characterized by an X’Pert PRO X-ray diffraction instrument (XRD) with Cu Kα as the irradiation medium. The scanning scale was 10° to 90°, and the scan speed was 0.27°/s.

### 2.5. Scanning Electron Microscopy

Microstructural observation of the coated specimens was conducted using a scanning electron microscope (SEM, JSM-6460, JEOL Ltd., Mitaka, Japan). The equipment had a 20-kV tungsten electron emission tip.

## 3. Results and Discussion

### 3.1. Coating Crystalline Structure

**Figure 1:**
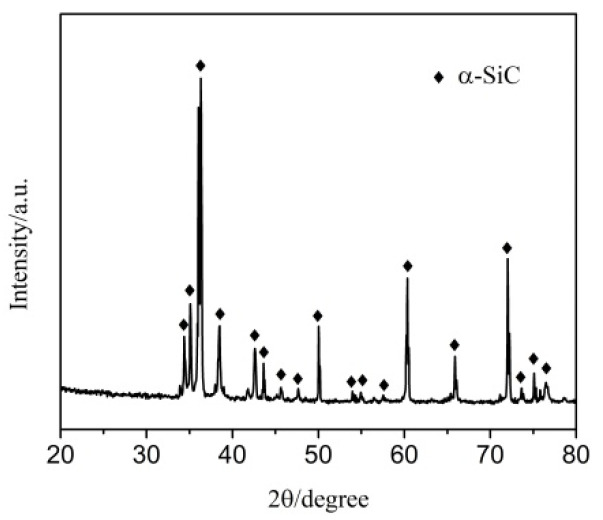
XRD pattern of the SiC coated C/C sample before oxidation.

Figure 1 shows the XRD pattern of the reactively sintered coating. The diffraction peaks match well with the characteristic peaks of α-SiC (JCSPD 01-089-3166). This result indicates that the coating is in the α-SiC phase, which is considered a high-temperature polytype that needs a relatively high temperature to grow [35]. Therefore, the coating has good high-temperature stability.

**Figure 2:**
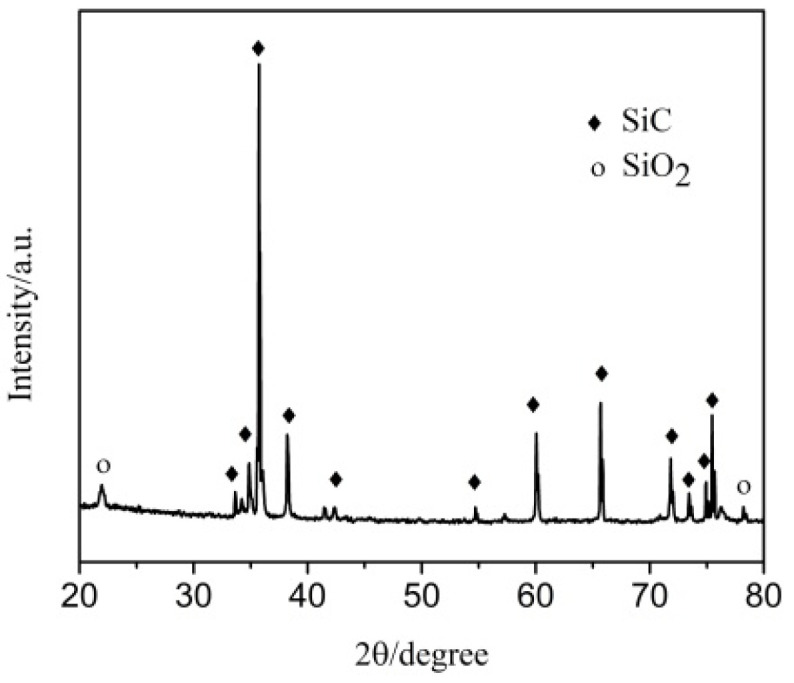
XRD pattern of the SiC coated C/C sample after oxidation at 1773 K in air for 20 min.

After oxidation, the coating is composed of SiO_2_ and SiC, for which the typical diffraction peaks are shown in Figure 2. This result indicates that SiO_2_ is formed from the reaction of SiC and O_2_ after oxidation at 1773 K in static air for 20 min.

### 3.2. Crack Healing Behaviour

**Figure 3:**
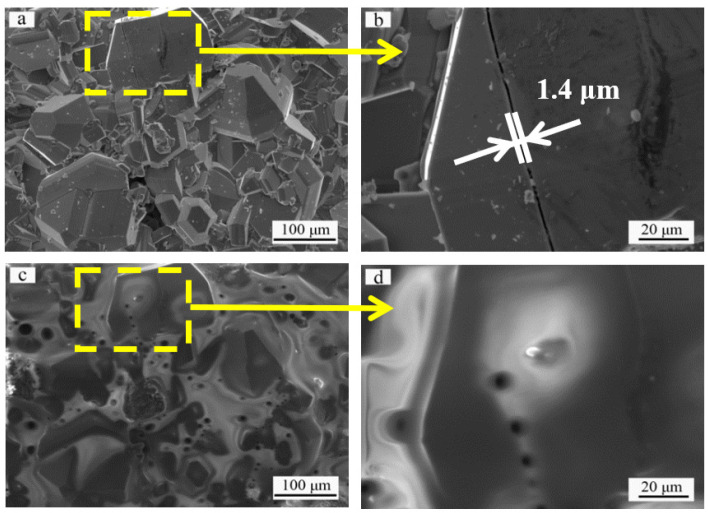
Surface morphologies of the SiC coating: (**a**) original surface before oxidation, (**b**) magnified image of the selected area in (**a**) showing a typical crack with 1.4 μm in width, (**c**) top view of the same area in (**a**) after oxidation, (**d**) magnified image of the selected area in (**c**) showing the healing of the crack.

To evaluate the healing ability of microcracks at high temperature, specimens with several types of microcracks of different widths (crack width < 3 μm, 3 μm < crack width < 7 μm and crack width > 7 μm) were selected to undergo oxidation at 1773 K in static air for 20 min; subsequently, the width of the oxidized microcracks was carefully compared against that of the original microcracks. Figure 3 depicts the surface morphology of the sintered SiC ceramic coating before and after oxidation. Figure 3a clearly shows a microcrack penetrating the SiC crystalline grain on the sintered coating surface. To observe the microcracks in detail, a magnified image (of the area marked in Figure 3a) is shown in Figure 3b. The crack width is measured to be 1.4 μm. The significant discrepancy in the CTE of the SiC ceramic coating (4.7 × 10^−6^ K^−1^ [36]) and C/C substrate (1.0 × 10^−6^ K^−1^ [37]) shows that microcracks were generated while the sample was cooling down from the coating preparation temperature. Figure 3c shows the SiC coating after oxidation at 1773 K for 20 min. The magnified image of the selected area in Figure 3c is presented in Figure 3d. Figure 3c shows the formation of a thin SiO_2_ glass layer. The viscosity of SiO_2_ at 1773 K is 1.91 × 10^9^ Pa s. The high fluidity of SiO_2_ leads to crack sealing [23]. Comparing Figure 3d against the morphology of the sintered SiC coating in Figure 3b shows the crack has disappeared, indicating that the crack in Figure 3b has completely healed.

**Figure 4:**
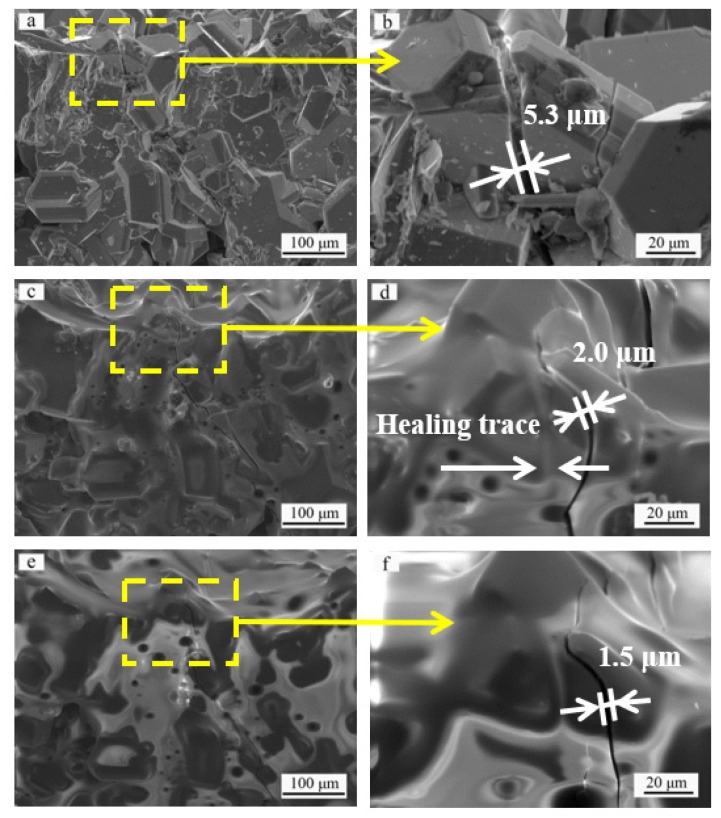
Surface morphologies of the SiC coating: (**a**) original surface before oxidation, (**b**) magnified image of the selected area in (**a**) showing a typical crack with 5.3 μm in width, (**c**) top view of the same area in (**a**) after oxidation for 20 min, (**d**) magnified image of the selected area in (**c**) showing the healing of the crack, (**e**) top view of the same area in (**a**) after oxidation for 40 min, (**f**) magnified image of the selected area in (**e**) showing the healing of the crack.

Figure 4 displays the surface microstructure of a SiC ceramic coating with a crack between 3 and 7 μm wide before and after oxidation. The topography of the sintered coating shown in Figure 4a is similar to that in Figure 3a. The ceramic coating is dense and has formed via aggregation of polygonal crystalline grains. A wider microcrack than that shown in Figure 3a appears in the coating. The magnified image (Figure 4b) shows that the crack is approximately 5.3 μm in width. After the sample has been oxidized at 1773 K for 20 min (Figure 4c), the original crack completely heals with a healing trace, but a new crack with a width of approximately 2 μm appears (Figure 4d). Figure 4e shows the surface morphology of the SiC coating oxidized at 1773 K for 40 min. Compared with the ceramic coating oxidized at 1773 K for 20 min, the SiC coating shown in Figure 4e has been further oxidized, and more SiO_2_ covers the ceramic coating surface. However, the morphology and the size of the crack in Figure 4f are different from those of the crack shown in Figure 4d, suggesting that the 2-μm wide crack (Figure 4d) has healed. The newly generated crack has narrowed (to a width of 1.5 μm), which may indicate that the residual thermal stress in the domain was decreased after oxidation of the sample at 1773 K for 40 min.

**Figure 5:**
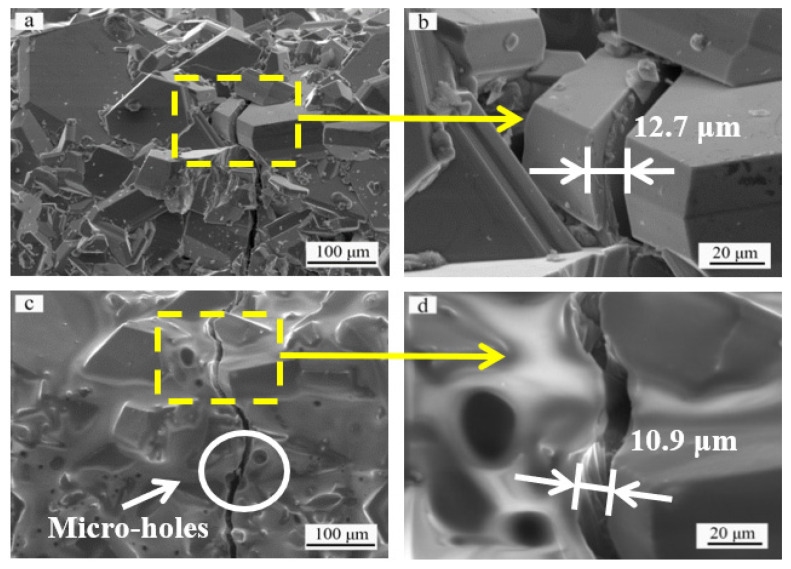
Surface morphologies of the SiC coating: (**a**) original surface before oxidation, (**b**) magnified image of the selected area in (**a**) showing a typical crack with 12.7 μm in width, (**c**) top view of the same area in (**a**) after oxidation, (**d**) magnified image of the selected area in (**c**) showing the healing of the crack.

Figure 5a displays the surface morphology of the sintered SiC ceramic coating with a crack more than 7-μm wide. Further observation (Figure 5b) shows that the crack width is approximately 12.7 μm. The morphology of the SiC coating surface after oxidation for 20 min is shown in Figure 5c,d. Based on observation of the two half micro-holes at the two sides of the crack (shown in Figure 5c), it is speculated that the 12.7-μm wide crack has healed during the oxidation process. The escape of gas oxidation products from the SiO_2_ glass layer creates micro-holes. As the coating undergoes a thermal cycle between 1773 K and room temperature, a crack forms on the oxidized coating surface (Figure 5c). The crack width is approximately 10.9 μm. Similar to the cracks with widths of approximately 5.3 μm and 1.4 μm, the crack with a large width of approximately 12.7 μm has been healed by oxidation at 1773 K in air for 20 min.

The following conclusions are drawn from the aforementioned results and analyses. Both the original crack that was formed during the coating preparation process and the newly produced crack that was generated while the sample cooled down from the high oxidation temperature healed perfectly during the oxidation process. Note that cracks with widths below 12.7 μm healed effectively, suggesting that coating failure does not result from these cracks.

### 3.3. Failure Behaviour of the SiC Coating

To investigate the failure of the SiC coating, isothermal oxidation was performed at 1773 K in static air. The probable chemical reactions [38] are listed below.

2SiC (s) + 3O_2_ (g) → 2SiO_2_ (l) + 2CO (g) (1)

SiC (s) + 2O_2_ (g) → SiO_2_ (l) + CO_2_ (g) (2)

2SiO_2_ (l) + SiC (s) → 3SiO (g) + CO (g) (3)

C (s) + O_2_ (g) → CO_2_ (g) (4)

2C (s) + O_2_ (g) → 2CO (g) (5)

**Figure 6:**
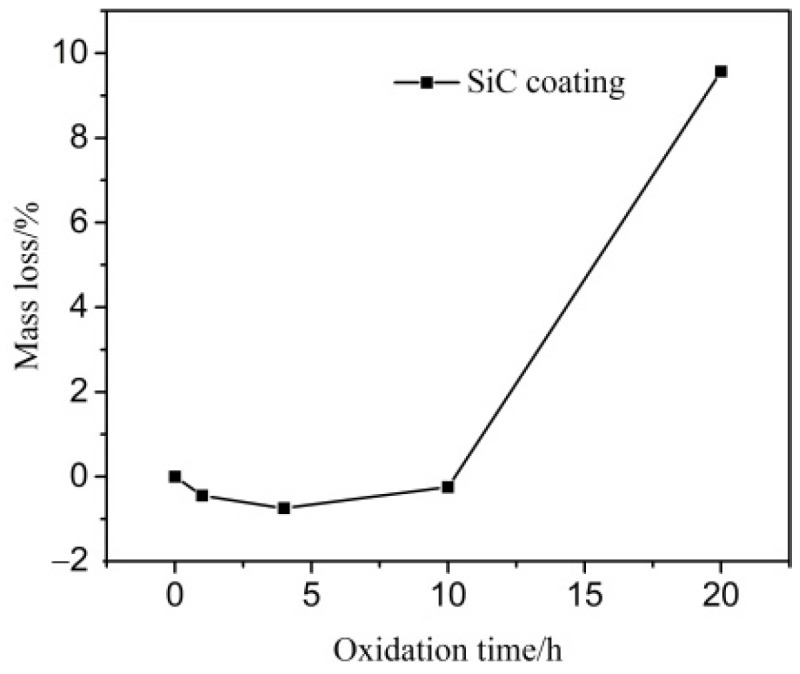
Oxidation curve of the SiC coating at 1773 K.

Figure 6 shows the mass-gain percentages of the coated specimen are 0.4% and 0.7% after oxidation for 1 h and 4 h, respectively. After being oxidized for 20 h, the mass-loss percentage of the coated specimen is 9.5%. Inspection of Reactions (1)–(5) shows that the mass gain results from the oxidation of SiC (Reactions (1) and (2)). The mass loss results from the release of the gas produced by oxidization of the C/C substrate (Reactions (4) and (5)).

**Figure 7:**
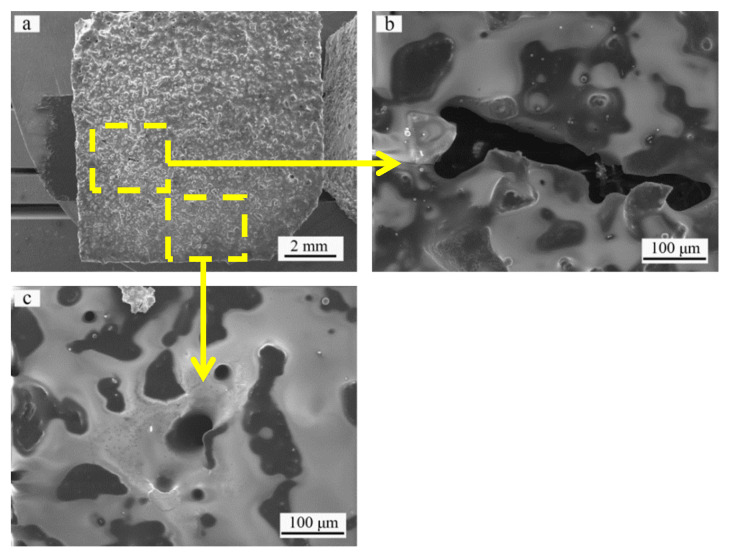
Surface morphology of SiC coating: (**a**) after oxidation at 1773 K in air for 20 h; (**b**,**c**) are the magnified SEM images of the selected area in (**a**).

The surface morphology of the SiC ceramic coating after 20 h of oxidation is presented in Figure 7. Different defects can be found in the coating, such as microcracks, holes and spallation. The selected area in Figure 7a is magnified in Figure 7b,c. Exfoliation and holes in the coating can be observed. Volatilization of the gas oxidation products from the melt SiO_2_ glass layer has created open holes. Hence, spallation and open holes facilitate oxygen diffusion, resulting in severe oxidization of the C/C substrate.

**Figure 8:**
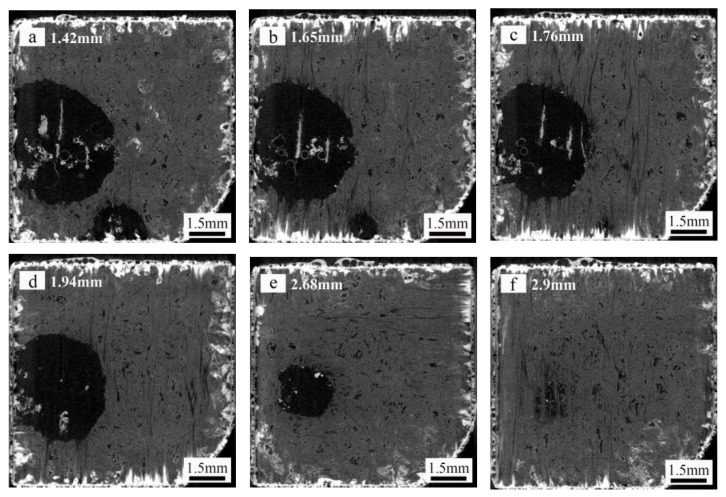
Virtual tomographic slices for the coating specimen of the Figure 7a.

X-ray computed tomography was performed to visualize the failure of the SiC-coated specimen presented in Figure 7a. A series of representative virtual tomographic slice images with different depths from the top surface of the coated specimen are shown in Figure 8. In Figure 8a, there are three clear phase contrast areas. Considering these images in conjunction with the characteristics of the C/C substrate, SiC coating and pores after oxidation, it can be inferred that the C/C substrate, SiC coating and pores correspond to the grey, white and black areas in the virtual tomographic slices, respectively. Two clear regions with oxidation damage (black regions) are observed in the C/C substrate (grey region) at a depth of approximately 1.42 mm. As the depth increases, the black regions gradually shrink. The small black region at the bottom of Figure 8a is barely discernible in the corresponding area of Figure 8c. The large black region presented on the left of Figure 8a has almost disappeared in Figure 8f. With a further increase in specimen depth, the black regions in the virtual tomographic slice images decreases noticeably, suggesting a decrease in the extent of oxidation in the substrate.

**Figure 9:**
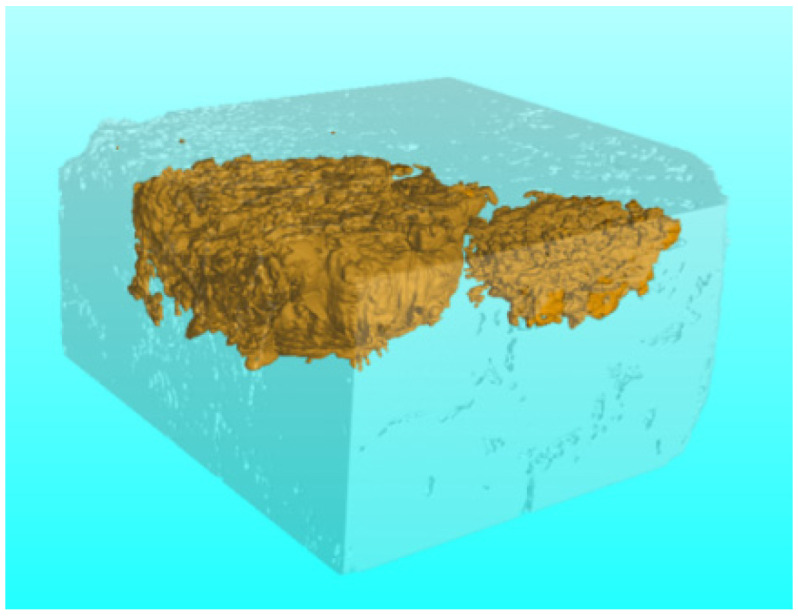
3D-reconstructed for the coating specimen of the Figure 7a.

X-ray computed tomography can also be used to determine the sites and morphology of oxidation damage. Figure 9 is a three-dimensional reconstruction of the coating specimen presented in Figure 7a, showing the appearance of two oxidation voids (yellow region) in the specimen. The voids in Figure 9 are almost semi-spherical, indicating that the failure sites are located at the upper surface of the specimen. The three-dimensional reconstruction image of the failure site in the SiC coating is consistent with the scanning electron micrographs. The voids in the left and right regions occur at depths of approximately 2.9 mm and 1.76 mm, respectively. Thus, X-ray computed tomography measurements facilitate identification of crack healing and the failure sites in the coating observed using SEM.

### 3.4. Failure Mechanism of the Coated Specimen

**Figure 10:**
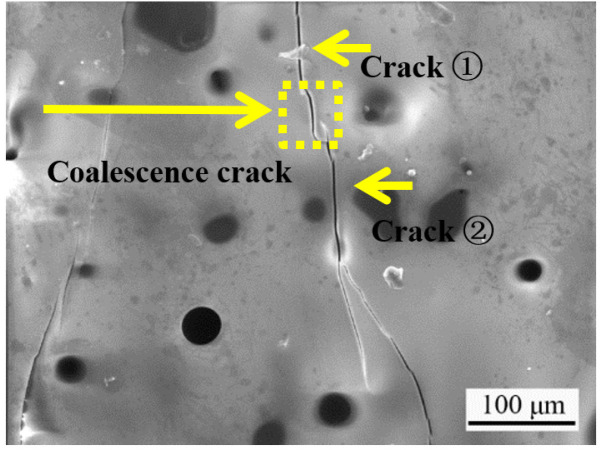
Tensile coalescence mode of crack coalescence.

**Figure 11:**
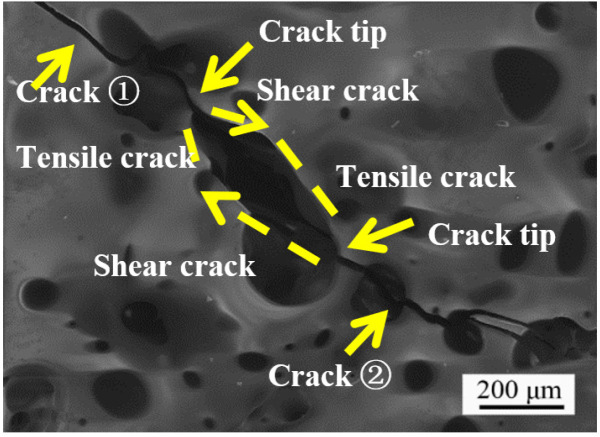
Mixture coalescence mode of crack coalescence.

The numerous studies about initiation, propagation and coalescence of crack leading to failure of rock or rock-like material have been reported [39,40,41,42]. However, studies have not been performed on a SiC-ceramic coating. The SiC ceramic is a typical brittle material. Investigating the initiation, propagation and coalescence of cracks is important for elucidating the failure mechanism of the ceramic in antioxidation coating applications. The CTE of the SiC coating is three times higher than that of the C/C substrate. While cooling down from the high oxidation temperature, the coating is subjected to residual tensile stress. Cracks are initiated when the local residual stress exceeds the strength of the brittle ceramic coating. Two types of cracks are usually observed in the coating after oxidation: tensile and in-plane shear cracks. If a tensile stress is exerted vertical to the crack surface, a tensile crack appears. The resulting displacement occurs in the direction of the tensile stress. If an in-plane shear stress is exerted parallel to the crack surface, an in-plane shear crack appears. The resulting displacement is in the direction of the in-plane shear stress. When the residual stress reaches a well-defined value, the crack begins to have a reciprocal effect on the surrounding cracks, resulting in crack coalescence and ultimately failure of the ceramic coating [43]. Coalescence is defined as the linkage of two cracks and occurs at the intersection of tensile and/or in-plane shear cracks [44]. The following cases of coalescence are found for the specimen coating after oxidation. In Figure 10, tensile coalescence occurs between crack ① and crack ②. Figure 11 shows the mixture mode of crack coalescence. The coalescence of tensile and in-plane shear cracks occurs at the tips of crack ① and crack ②. As the residual stress increases, coalescence occurs via the linkage of the tensile crack and in-plane shear crack between crack ① and crack ②.

**Figure 12:**
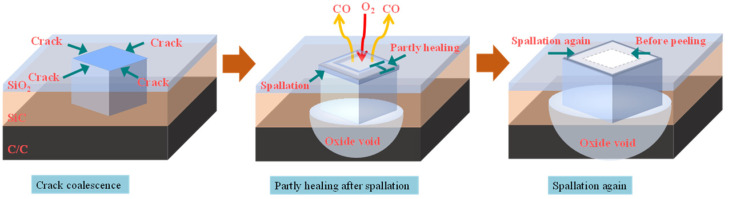
Schematic diagram of the failure process for SiC coating.

The following failure process of the SiC coating is inferred. As shown in Figure 12, spallation is generated during the thermal cycle after crack coalescence. The oxidation product SiO_2_ heals the crack during oxidation, but the spallation region is not completely healed. Oxygen can diffuse from the partially healed spallation site into the specimen and react with the C/C substrate. Reaction (5) shows that an oxygen molecule diffusing into the specimen will produce two carbon monoxide molecules, leading to an increase in the internal gas pressure of the coating. Carbon monoxide escapes from the weak region in the coating. When the outward diffusion of carbon monoxide equilibrates with the inward diffusion of oxygen, the size of the spallation region remains unchanged. The spallation region increases in size in the course of the thermal cycle during oxidation. Therefore, it can be concluded that the failure of the coating is caused by spallation and open holes.

## 4. Conclusions

SiC ceramic-coated C/C specimens were fabricated by PC, and specimen failure induced by oxidation at 1773 K in static air was investigated using X-ray computed tomography. Both original cracks that formed during the coating preparation process and newly generated cracks that formed while the specimens cooled down from the high oxidation temperature were healed perfectly because of the formation of an SiO_2_ glass during oxidation at 1773 K in air for 20 min. X-ray computed tomography images showed that spallation resulting from crack coalescence and open holes provided channels for oxygen to penetrate the specimen. Increasing the oxidation duration caused enlargement of the spallation region, thereby resulting in the oxidation failure of the coated specimen.

## Data Availability

Not applicable.
